# Development of a Pilot Specialist Dementia Nurse at a Tertiary Victorian Hospital

**DOI:** 10.1177/14713012251364329

**Published:** 2025-08-13

**Authors:** Rebecca Leahy, Dina LoGiudice, Joanne Tropea, Fleur O’Keefe, Sharne Donoghue, Jenna Dennison, Alissa Westphal, Aaron B. Wong, Dayalini Kumarasamy, Kathryn A. Ellis, Nicola T. Lautenschlager

**Affiliations:** 1 Allied Health Department, 90134 Royal Melbourne Hospital, Parkville, VIC, Australia; 2 Department of Medicine – Royal Melbourne Hospital, 2281 University of Melbourne, Parkville, VIC, Australia; 3 Wellbeing in Ageing Team, Department of Psychiatry, 2281 The University of Melbourne, Parkville, VIC, Australia; 4 Department of Aged Care, 90134 Royal Melbourne Hospital, Parkville, VIC, Australia; 5 Faculty of Medicine, Dentistry and Health Sciences, 2281 University of Melbourne, Parkville, VIC, Australia; 6 Royal Melbourne Hospital Mental Health Services, 90134 Royal Melbourne Hospital, Parkville, VIC, Australia

**Keywords:** dementia, nurse, pilot, hospital, post-diagnosis, health service

## Abstract

The pathway to a diagnosis of dementia and post-diagnostic support can be complicated for people living with dementia and their support networks, leading to delays in care. This paper describes the development and subsequent pilot of a specialist dementia nurse role named the Dementia Liaison Officer (DEMLO) within a tertiary Victorian hospital. The aim of the DEMLO was to identify and address gaps in dementia care. This pilot is based on the principles of the National Comprehensive Dementia Centre framework. Mapping of current outpatient diagnostic and post-diagnostic services for people with suspected cognitive impairment was conducted to identify gaps in care. Interviews with 25 hospital staff were also conducted to gain further perspectives on barriers to diagnostic and post-diagnostic care and inform the development of the DEMLO role. Several challenges to diagnostic/post-diagnostic support were identified, including the perception that waitlists were long, confusing referral criteria, and complex referral processes. The DEMLO was subsequently developed and trialled within the Geriatric Evaluation and Management (GEM) outpatient service. Three key functions were implemented: reviews of eligibility criteria for patients with cognitive impairment on the GEM clinic waitlist, introduction of a pre-clinic comprehensive geriatric assessment for GEM clinic patients and the introduction of a dementia post-diagnostic support service for all patients across the hospital. Challenges to developing and sustaining the pilot included limited timeframe, difficulty with integration and limited funding. Despite this, the pilot was well received, with 141 patients referred. The introduction of a nurse-led dementia intervention has resulted in increased person-centred care that encompasses pre-diagnostic and post-diagnostic support for people living with dementia. Evaluation of the pilot is ongoing.

## Introduction

It is estimated there are currently more than 421,000 people living with all forms of dementia in Australia, and this number will rise to 812,500 by 2054 (Dementia Australia, 2024). In Australia, dementia is the second most common cause of disability, with a predicted annual cost of $36 billion by 2056 (Australian Institute of Health and Welfare, 2024; Brown et al., 2017; Dementia Australia, 2024). Globally, there is a push for more accurate and timely diagnosis of dementia, as this maximises the opportunity for patients and families to gain access to appropriate education, therapies, clinical care pathways and research trials. It is also important for opportunities to implement advanced care directives, Enduring Power of Attorney (EPOA) and other services (Panegyres et al., 2016). Despite this, many people living with dementia and their families liken the process of receiving a diagnosis and treatment to trying to navigate their way through a hundred different doors (National Comprehensive Dementia Centre Network, 2022). At an international level, much research effort has gone into identifying the barriers and facilitators to equitable and accessible dementia care, with results suggesting that services are limited by poor care coordination and unclear access procedures (Bernstein Sideman et al., 2022; Sorrentino et al., 2025). Socio-economic and cultural factors also significantly impact engagement and quality of care, not only at the access point to services, but whilst navigating the entire system (Seeher et al., 2023; Watson et al., 2021). Access to dementia diagnosis and post-diagnostic care in Australia mirrors these findings, with geographical location, stigma, cultural background, disjointed referral pathways, lack of multi-disciplinary support and non-standardised diagnostic criteria all identified as barriers to appropriate dementia care (Australian Government, 2017; Giebel et al., 2023).

The process for receiving a diagnosis and accessing post-diagnostic support varies greatly depending on location and the clinical setting (Robinson et al., 2015). In higher-income countries, screening for dementia in groups at high risk is common and is often performed by primary care clinicians (Jones et al., 2024). However, diagnosis requires a range of measurements, including extensive cognitive testing, functional assessments and when applicable, brain imaging and biomarker analysis, and access to these vary greatly worldwide (Parra et al., 2019). Similarly, post-diagnostic support is highly variable, dependent on health care setting (hospital vs. community) and is often untailored to the patient or carer (Jeon et al., 2024). In Australia, an initial access point is often a general practitioner (GP), where a patient could then be referred to an outpatient specialist service (Ng & Ward, 2018). Alternatively, they may be identified in a hospital, where they can be assessed by inpatient services, referred to outpatient geriatric, psychiatry or neurology services, or discharged to be followed up by their GP. Each of these pathways can lead to different timelines for diagnosis and follow up care and are also limited by consumer and health professionals’ understanding of each service (Iliffe et al., 2003). Other barriers to accessing care include delays in family members reporting symptoms of cognitive impairment, delays between initial appointments and obtaining a diagnosis, and delays in accessing support following diagnosis (Claveau et al., 2018; Walker et al., 2018). Another obstacle to an accurate diagnosis occurs when a patient who was admitted to hospital for a different reason (such as a fall), and whilst a diagnosis of dementia may have been suggested, it was not communicated to the patient or their family (Walker et al., 2018).

Patient and carer experiences in diagnostic settings in Melbourne demonstrated that one third of participants reported dissatisfaction with their overall dementia diagnostic experience. Participants in this study specifically described problems with the level of information that was provided, and the lack of opportunity for further support (Ng et al., 2021). Further research with Australian clinicians working in memory clinics paints a similar picture, emphasising the need for a single point of support (“one stop shop”) for people living with dementia and their carers, and more funding for memory clinics to implement more effective cognitive interventions and high-quality training (Steiner et al., 2020). This research mirrors global recommendations for integrated dementia support services across all stages of prevention, diagnosis and post-diagnostic support, thereby allowing the sharing of resources and collaboration with people living with dementia and their carers (Wang et al., 2022).

From this data emerges a need for a streamlined referral process and greater access to clinical care, post-diagnostic support services and research trials. The National Comprehensive Dementia Centre (NCDC) model of care described a comprehensive and collaborative approach to dementia services aimed to target these gaps in Australia. This included the introduction of a dementia-specific clinical nurse coordinator (National Comprehensive Dementia Centre, 2022). The role of a nurse-led dementia service has received strong interest from GPs and consumers (Pond et al., 2021). Particularly, evidence from the UK suggests that specialist dementia nursing services (Admiral Nurses) that work closely with patients and families in a primary care role or within hospital settings may speed up diagnosis. This assists in reducing pressure on GPs and specialists, and increases patient and carer satisfaction (Aldridge et al., 2020; Hayhoe et al., 2016; Piercy et al., 2018). International models of collaborative care have been successfully implemented and evaluated. These models have been shown to be cost effective and beneficial for improving outcomes for people with dementia and their carers (Panlilio & Evertson, 2023; Possin et al., 2019).

### The National Comprehensive Dementia Centre (NCDC) Framework

The NCDC vision is to implement comprehensive dementia hubs across Australia that integrate diagnostic and clinical care, research, clinical education and support services for people with dementia and their families. In collaboration with the University of Melbourne, a pilot project of a specialist dementia nurse (one of the key features of the NCDC model) was proposed to trial within a tertiary hospital in Victoria. This pilot had a project group that consisted of two academic and two executive members of the university, five clinical and two executive members of the hospital, and representatives from Dementia Australia and the Australian Dementia Network Clinical Quality Registry. The design of the nurse role was a decision between the project group, which was ultimately approved by the Dean from the University and the CEO of the hospital. It was also endorsed by the NCDC Steering Committee.

### Aims/Objectives

The aims of this project were:

1. To identify gaps in current services for people with cognitive impairment requiring further diagnosis.
2. To identify gaps in current services for people with dementia requiring post-diagnostic care.
3. To assess barriers and enablers to implementing a specialist nurse role with consideration to the context and timeframe.
4. To develop and trial a specialist dementia nurse role that addresses the gaps identified and can be operationalised with consideration to the barriers and enablers of the role.

## Methods

### Hospital Setting

The chosen hospital site was a Victorian tertiary public hospital, which offers free services for all patients under the Australian Medicare scheme. The Cognitive Dementia and Memory Service (CDAMS) was initially recommended as the chosen service to implement a specialist dementia nurse role. CDAMS is a specialist Victorian public health initiative with 22 clinics across Victoria. The service provides multidisciplinary care for older adults generally over the age of 65, experiencing memory loss, or changes to their thinking (cognition). CDAMS is a time-limited diagnostic service that also provides advice and education to patients and families; however, it does not provide ongoing care.

### Participants

Participants for this study included 25 internal hospital staff members, which included 17 clinical staff, four executives and four administration staff. Participants were selected via purposive sampling, with the aim of gathering a wide range of perspectives from staff working in different aspects of dementia care. Administrative and clinical staff were not included to participate in the study if they did not work with/within dementia services. Prospective participants were suggested by key staff of the project and approached for participation via email. Informal interviews were set up in either a face-to-face or online format. Two senior nurses were employed to undertake the role of the specialist dementia nurse, in addition to the role of research officers to complete the interviews and hospital mapping.

### Procedure

#### Aims 1-2

An initial review and audit of the existing outpatient service delivery and infrastructure was undertaken. This was conducted in collaboration with clinical and administrative staff at the hospital, in addition to reviewing available eligibility criteria documents for all outpatient clinics that offer services for patients with cognitive impairment (five current documents and two outdated documents). Hospital mapping was completed, and focused on the internal (e.g., inpatient wards) and external (e.g., GPs) pathways into outpatient services, and addressed referral processes, triaging, assessment, management and follow-up of patients with suspected cognitive impairment. One-on-one informal interviews were conducted with staff members, with interview questions co-designed by the two specialist dementia nurses (shared role of 1 FTE (full time equivalent)) who were funded by the pilot project to undertake the nursing role (renamed the Dementia Liaison Officer (DEMLO)) and conduct the interviews. These specialist nurses were employed as Grade 5 Clinical Nurse Coordinators, and both had several decades of clinical nursing experience, in addition to postgraduate qualifications (Masters degrees) in clinical nursing and aged care/dementia. One of the nurses was also an endorsed Nurse Practitioner.

Informal interviews consisted of information gathered during brief non-clinical meetings that were semi-structured. Interviews were between 30 min–60 min in length, and were not recorded or transcribed, however handwritten notes were taken. Whilst one of the nurses was an employee of the hospital, they were not directly acquainted with any of the participants. The second nurse was new to the hospital and therefore was not known to participants. Questions were developed beforehand by the study team to potentially ask during the interviews, with the goal of addressing any gaps noticed in the eligibility documents that were reviewed. The questions were as follows:

1. What outpatient services assess people with suspected dementia (or with cognitive impairment)?
2. What are the eligibility criteria for each outpatient service? What are the exclusion criteria?
3. How can health practitioners refer to the service (e.g., by filling in a template, sending an email etc.)?
4. How are prospective patients triaged?
5. Once accepted into the service, what is the assessment and management process? Which healthcare professionals are involved?
6. What, if any, ongoing follow-up or support do patients receive?
7. At what stage do they get discharged?
8. How is your service currently delivered, and do you collect data on your current pathways?
9. How is information shared between different departments and stakeholders?

Concurrently, further informal interviewing was conducted to gain perspectives on the staff perceived barriers for patients with suspected cognitive impairment accessing appropriate diagnostic and post-diagnostic support. Questions were asked surrounding:

• Potential barriers to referring patients to outpatient services.
• Challenges with triaging/cause of delays to triaging.
• Impact of delays and waitlist times/time to assessment.
• Assessment and management processes.
• Discharge and follow-up processes.

To assist with categorising and understanding the types of issues raised in the interviews, we used the six dimensions of quality of care as a guide (from Australian Commission on Safety & Quality in Health, 2009), acknowledging that the development of strategies in response to these issues requires clearly defined goals and actions. These dimensions were used as a priori themes for thematic analysis.

#### Aims 3 and 4

In collaboration with the NCDC Steering Committee, the pilot project’s key staff formed the concept of a DEMLO to pilot within a Victorian hospital. The type of nursing service provided was designed based on the identified gaps in current care processes, and the best use of the two nurses specialised skills in dementia and aged care services. The initial proposed model of the specialist DEMLO was discussed with participants, with the goal of operationalising the model to suit the hospital’s current processes. No consumers were involved in the design of the model; however, the design was informed by the NCDC model of care which involved extensive consumer consultation. Topics discussed included:

• What areas of the hospital are in most need of further support?
• What services can the specialist dementia nurse provide, beyond what is already being provided?
• What will be the impact of the specialist dementia nurse on existing staff roles?
• How will the specialist dementia nurse’s work be documented in the Electronic Medical Record (EMR)?
• How will patients be referred to the service?
• How will the service be advertised and/or phased out if ongoing funding is not secured?
• How can the service remain cost-neutral to the hospital?

Discussion and refinement of the DEMLO role was conducted over six months and completed concurrently with hospital mapping and other informal interviews. Notes from the mapping and interview process were amalgamated, and the research team identified key themes and issues to address when implementing the DEMLO role.

### Data Analysis

All information collected from the interviews was analysed using narrative thematic analysis (Crossley, 2000; Kohler Reissman, 2002), with a-priori themes identified. Descriptive analyses of triage processes, clinic-specific processes and gaps in care was undertaken, and data were synthesised to map existing diagnostic and post-diagnostic pathways.

## Results

The results of this study are described as follows:

• Identified gaps in current care practices via a review of referral, diagnostic and post-diagnostic pathways for people with suspected cognitive impairment (and how these pathways differ from each other), and information from internal stakeholder interviews.
• Barriers and enablers to the introduction of the DEMLO role.
• Reconfiguration of the role in response to identified barriers/enablers.

### Gaps in Diagnostic and Post-Diagnostic Care

**Figure 1.**
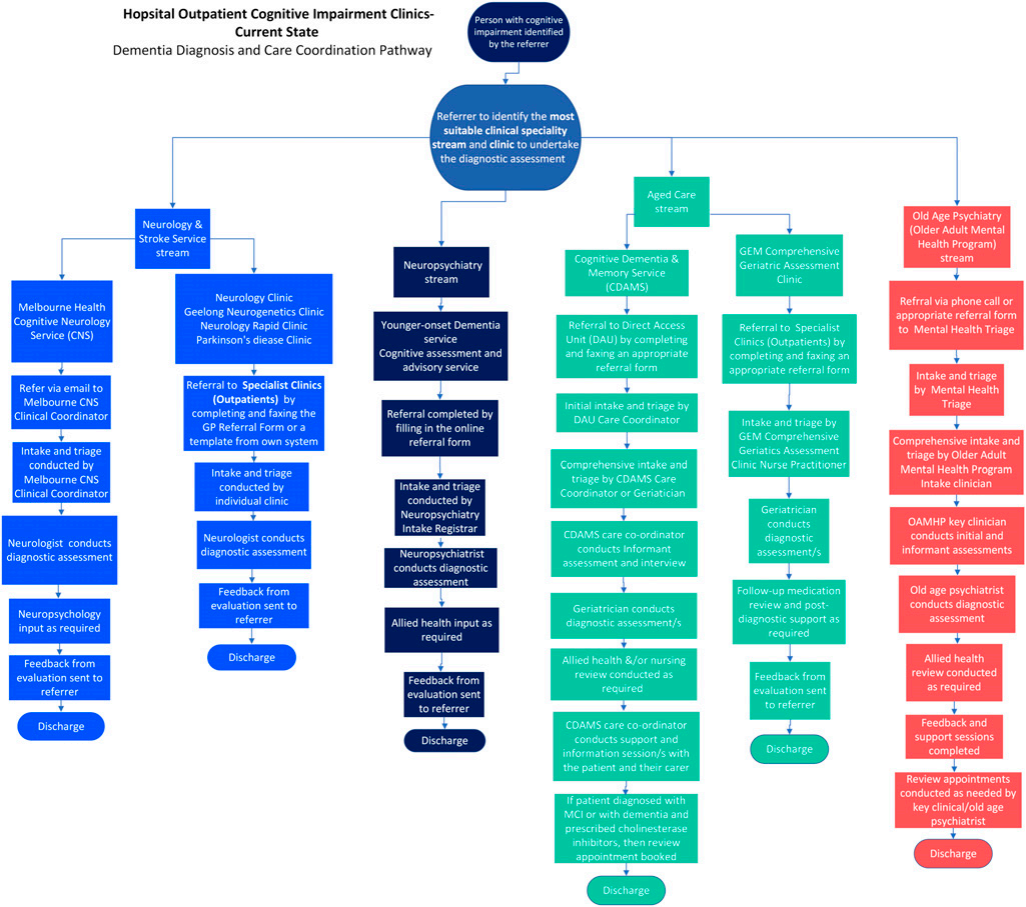
Mapping of the Pathways to Diagnostic and Post-Diagnostic Care in Outpatient Cognitive Clinics

Figure 1 demonstrates the main outpatient diagnostic pathways that patients with suspected cognitive impairment can be referred to within the hospital.

#### Services Available for Referrals

Four main referral pathways/services were identified:

i. Neurology and Stroke Service
ii. Neuropsychiatry
iii. Aged Care
iv. Old Age Psychiatry (Aged Mental Health)

The Neurology and Stroke Service, and Aged Care pathways are further broken down based on the goals of the referral (specifically related to cognition vs. other needs). Referrals can be made by a variety of processes, including email, filling out a referral form and faxing, online referral system and phone calls.

#### Triage

Triage processes for each service vary greatly. Some services have dedicated intake and triage teams (e.g., Direct Access Unit for CDAMS), whereas others may have triage completed by one individual (e.g., Nurse Practitioner for GEM clinic).

#### Assessment and Ongoing Management

All outpatient services offer diagnostic assessments to determine the cause of cognitive impairment. Allied health input varies across services, with some offering multidisciplinary support (e.g., CDAMS, Neuropsychiatry, Old Age Psychiatry), and some being limited to neuropsychological support (e.g., Cognitive Neurology). The GEM clinic only offers medical and nursing support. Services that offered post-diagnostic support were CDAMS, which is limited to one support session, and the Old Age Psychiatry Service.

#### Factors Impacting Quality of Care

Engagement with internal stakeholders (administrative and clinical staff) revealed several factors that impact people with cognitive impairment from accessing and receiving care from the above mentioned outpatient services. These are summarised in Table 1.

**Table 1.** Perceived Gaps and Barriers to Accessing Outpatient Diagnostic and Post-Diagnostic Care by Quality of Care Dimensions

Quality of care dimensions		
Access	Efficiency	Appropriateness (of referrals)
Some felt that the CDAMS clinic criteria is too strict and/or that the waitlist is very long.	There are too many different referral modes dependent on the clinic.	Many outpatient clinics offer diagnostic services related to dementia. Five clinics have dementia or cognition in their title, which can make choosing the appropriate service confusing for referrers.
Most services are considered “diagnostic” and do not provide ongoing post-diagnostic support.	Intake and triage staff estimate that 4 h per week of clinical assessment time is lost in duplicate intake and screening (due to multiple referrals for one patient).	Each of the cognition and/or dementia titled clinics have their own eligibility criteria. Most of the eligibility criteria are not published internally or externally.
Estimated 4–6 month wait list time for the GEM clinic due to high volume of referrals requiring Nurse Practitioner intake and triage	Staff report that confusion amongst external referrers (particularly GPs) has resulted in an ongoing high volume of out-bound calls to clarify patient suitability for clinic services.	
Patients get discharged too early from CDAMS – many have complex needs that require ongoing follow-up.	Procedural documents do not contain any information for internal staff regarding how any of these outpatient dementia clinics operate. There are different individual webpages for each service stream and clinic for external providers.	
Patients with delirium are rarely referred due to strict intake criteria. It is suspected they are referred to private geriatricians instead, or for follow-up with their GP.	Sharing of information between clinics is poor.	

*Note. A*s cited by the Australian Commission on Safety & Quality in Health (2009).

### The Introduction of the DEMLO Role and the Barriers/Enablers

In the initial conceptualisation of the pilot, it was intended for the DEMLOs to be co-located in the CDAMS clinic, due to the potential of providing greater access to ongoing post-diagnostic support and a greater opportunity to engage with GPs. Positioning in the CDAMS clinic would also allow for ease of integration in the Electronic Medical Record (EMR), making the referral process to the DEMLOs identical to referral system of the rest of the hospital. The initial aim of the DEMLO role was to oversee and streamline referrals, coordinate care and provide greater access to post-diagnostic supports, ultimately providing a “one stop shop” for people living with dementia, and their carers. Consultation with participants revealed the following barriers and enablers to the initial service model.

#### Barriers

Given the operational barriers to implanting the DEMLOs in CDAMS, it was difficult to integrate the DEMLOs into the hospital without creating a standalone clinic, which was estimated to take a minimum of three months, and establishing a standalone nurse-led clinic was not feasible in the project timeframe. Operational barriers included:

• Altering the Electronic Medical Record (EMR) to allow for direct referrals to the DEMLOs was not possible during this timeframe.
• Following up patients for longer-term post-diagnostic support in CDAMS proved to be a challenge due to most patients being discharged following diagnosis (and once the appropriate medication review/information and support session was completed).
• Integrating the DEMLOs into alternative clinics was limited by funding schedules, such as the Medicare Benefits Schedule, which requires medical contact hours to outweigh nursing contact hours.

Other considerations included:

• If ongoing funding for the specialist dementia nurses was not able to be obtained, it would be impractical to advertise the service to external referrers such as GPs (as the service would need to discontinue).
• Utilising the DEMLOs for triaging would impact existing employees’ roles.
• Running a standalone nurse-led clinic would need to be cost neutral for the hospital, and was not feasible to set up in the timeframe.
• Patients and carers may not notice the impact of the DEMLOs if they worked “behind the scenes” with triaging and re-routing patients on waitlists, and a better use of their skills may be seen in a more “hands on” clinical role.

#### Enablers

The need for the DEMLOs was echoed amongst the majority of participants, and there was a high level of enthusiasm for the role. Many participants reported feeling uncertain about what to do when they identified suspected cognitive impairment in a patient who was an inpatient/receiving outpatient care for a different reason, and that having a central point to direct queries to would help solve this problem. The ability to provide people living with dementia and their families with a contact for further post-diagnostic support was also highly regarded, as this was recognised as a gap in current care processes.

Other enablers included:

• Large numbers of patients with suspected cognitive impairment across the hospital, which allow for the opportunity for the pilot to have a considerable impact on current care practices.
• Multiple specialist clinics to route referrals to, ensuring patients with suspected cognitive impairment receive care that is tailored to their needs.

### Reconfiguring the Dementia Liaison Officer (DEMLO) Role

**Figure 2.**
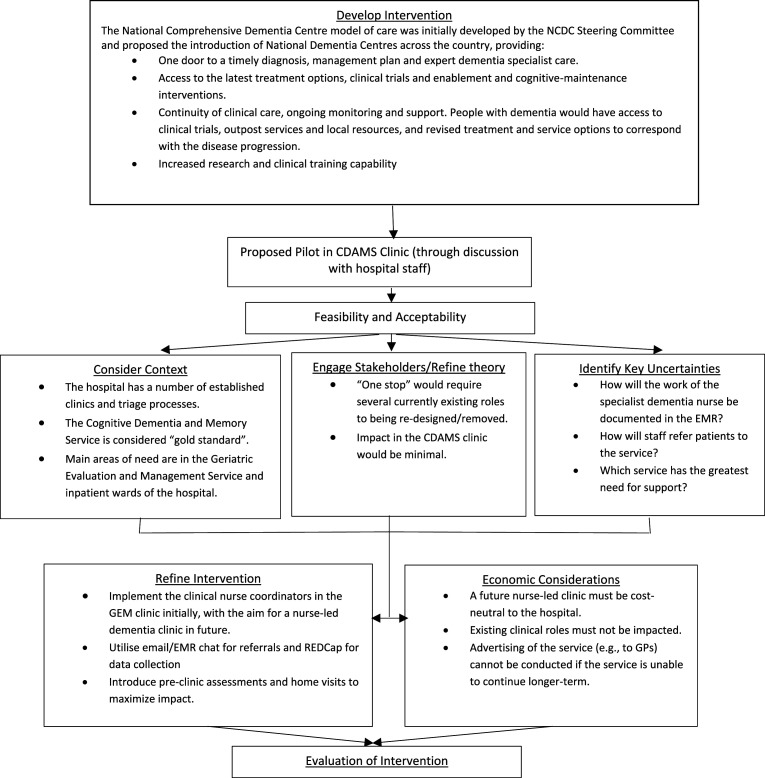
Flow Chart of the Reconfiguration of the DEMLO Role. *Note*. NCDC = National Comprehensive Dementia Centre; CDAMS = Cognitive Dementia and Memory Service; EMR = Electronic Medical Record; GP = general practitioner; GEM = Geriatric Evaluation and Management

After identification of the barriers and enablers to the DEMLO position, a reconfiguration of the role was required. Utilising the Framework for Developing and Evaluating Complex Interventions (Skivington et al., 2021) and the Consolidated Framework for Implementation Research (CFIR; Damschroder et al., 2022) a guide, an operational model of the DEMLOs was proposed (see Figure 2). With executive endorsement, the DEMLO role was chosen to be positioned within the existing outpatient Geriatric Evaluation and Management (GEM) clinic, rather than the CDAMS clinic. This Medicare Benefits Schedule (MBS) funded clinic provides specialist assessment and management by a team with medical and nursing expertise. Patients referred to the clinic are generally aged over 65 years and have complex multimorbidity encompassing medical, functional, and often cognitive issues requiring completion of a comprehensive geriatric assessment (CGA). Currently the outpatient GEM clinic receives approximately 300-330 new referrals per year.

After extensive consultation with the GEM outpatient clinic staff; the DEMLOs were able to create a model of care which included three keys services that operated within the GEM clinic. These were:

i. For new patients referred to the clinic, the DEMLO will review the referral to confirm the patient’s eligibility for review in the GEM clinic and the priority/urgency for this review.
ii. For patients accepted to the GEM clinic waitlist, the DEMLO will conduct a comprehensive nursing review (via telehealth or home visits) prior to their in-clinic appointment.
iii. A post-diagnostic support service was offered for all patients across the hospital. For all patients who recently received a dementia diagnosis, or where the need for support has been identified for an existing dementia diagnosis, the DEMLO will provide post-diagnostic information, education, care planning and co-ordination to the patient and their carer to help them manage this need.

The third service was advertised within the hospital, particularly within the Emergency Department, Acute Medical Unit, aged care wards and general medical wards with a focus on post-diagnostic/post-hospitalisation support. A further focus was directed at patients recently discharged with delirium (due to this being an exclusion criterion for access to several clinics) and patients involved in the Hospital Admission Risk Program (HARP). HARP is a service that provides support and care for people with chronic and complex health issues, with the goal of enabling them to better manage their condition in the community and reduce avoidable hospital admissions.

To facilitate a simple and timely referral process, referrals to the DEMLOs were made via direct GEM referrals (using a patient spreadsheet), EMR secure chat and via a purpose-built DEMLO email address. Data was collected and stored in a purpose-built REDCap database and transferred into the EMR as required.

### Current Implementation

Implementation of the DEMLO pilot began in April 2024, following approximately six months of hospital-mapping, program development and obtaining ethics approval to evaluate the pilot. The pilot was completed in October of 2024, with 141 patients in total referred to the DEMLO service. The results of the evaluation are expected to be available by later in 2025.

## Discussion

### Key Learnings

The National Comprehensive Dementia Centre Framework initially set out to establish one-stop clinical and research dementia hubs where the path to diagnosing and managing dementia is clear and easy to follow, and links through to cutting edge research are easy to find. This pilot project was the first step to creating a more integrated dementia service within a Victorian tertiary hospital. The DEMLOs were set up in a way that addresses current gaps within the hospital, particularly regarding referral clarification and post-diagnostic follow-up. Our project demonstrated that there are currently several specialist services and clinics related to the diagnosis and management of people with suspected cognitive impairment. The diversity of services allows for tailoring of care for patients requiring diagnosis and treatment. Whilst there are several benefits to the current pathway to a cognitive impairment or dementia diagnosis within the hospital, some barriers related to access, efficiency and appropriateness of referrals were identified by hospital staff. It was also identified that there is limited access to post-diagnostic support, and this is dependent on which clinic a patient is referred to. The introduction of the DEMLOs helped begin the process of addressing some of these gaps, however some challenges to the pilot project were encountered. The original conceptualisation of the specialist dementia nurse was difficult to operationalise and implement in the timeframe of the pilot project. This was mainly due to insufficient time allocation to manage the logistical and operational impact to the hospital, and a lack of appropriate funding for an ongoing service. These findings are in-line with other literature reporting barriers to implementing hospital-based interventions, which commonly include environmental factors (IT, workflow, etc.) and ease of integration (complexity, cost, suitability to system), among others (Geerligs et al., 2018; Zoutman & Ford, 2017). These difficulties are also replicated in other attempts to create comprehensive dementia models, with competing priorities and financial viability common barriers to implementing these services (Reuben et al., 2023).

The operational model to be utilised by the DEMLOs, as well as the associated potential outcomes, had to be reconfigured by the research team. Through extensive consultation with stakeholders and consideration of the processes suggested within the Consolidated Framework for Implementation Research (CFIR; Damschroder et al., 2022) and the National Institute for Health Research Framework (Skivington et al., 2021), several positive measures and systems were able to be established. In line with the implementation process domain outlined in the CFIR, we were able to create a DEMLO role that optimally integrated with existing hospital processes. We anticipate that the role of the DEMLO will have positive outcomes on consumer and staff satisfaction with dementia care.

### Limitations

The development of the DEMLO role had several limitations. The first was that this was a staff-designed role, with no consumer involvement in the reconfiguration and implementation of the service. Due to the limited timeframe of this project, the opportunity to seek consumer involvement was not feasible. Whilst the original NCDC business case involved extensive consumer consultation and the involvement of Dementia Australia, we recognise that the perspectives of consumers would have been very valuable in determining the areas of need for the pilot service.

Further, another limitation is that the opportunity to be involved in the development of the DEMLO role was limited to staff who were recommended by the project’s key organisers. Whilst the recommended staff members were key stakeholders within the hospital and very knowledgeable within their particular department, the development of the DEMLO lacked input from a wider range of staff members across all levels of experience.

The reduced timeframe of the pilot was also a limitation of this study. As the pilot was only able to be active for approximately six months, it was difficult to implement the required IT modifications, and had consequences for the advertising of the service across the hospital.

### Recommendations for Implementation of a DEMLO Role

From this project arises several recommendations for the development and implementation of a DEMLO or similar role in other health care services. First, would be to allow for a longer period of consultation with the service to identify gaps in care, the potential environmental and economical impact to the service, the ease of integration, IT and personnel issues and the long-term sustainability of a DEMLO role. Our consultation period lasted approximately six months, however there were still some outstanding issues with integration and sustainability that could have benefitted from further discussions before the pilot commenced. As discussed by (Dias et al., 2010), role ambiguity and relationships with staff are key to the implementation of nursing roles, and our DEMLOs would have benefitted from increased engagement with other staff to integrate the service with the rest of the hospital. As a result, we recommend allowing 9–12 months total for the development process, which is in line with a participatory action research approach outlined by Dehennin et al. (2023). Further, the pilot was only able to be implemented for a period of six months, which had consequences for the integration of the service at a hospital-wide level (e.g., difficulties with IT modifications, advertising was limited due to lack of an ongoing service). In future, implementations of a DEMLO or similar role would benefit from a pilot period of at least 12 months (Leon et al., 2011).

In addition, future developments of a DEMLO or similar role would benefit from discussions across all wards, clinics and individual teams working in dementia services to ensure greater coverage of potential gaps in care. This is particularly pertinent for future iterations of a National Comprehensive Dementia Centre framework (or similar comprehensive dementia frameworks), as identifying what currently works well (e.g., the Cognitive Dementia and Memory Service within this particular context), will be an important step for building adjunct and integrated services within an already well-established system. Having a framework or guideline to optimally begin integration of services would be beneficial to assist executive and clinical staff in managing changes, particularly within the context of the administering healthcare system.

Finally, increasing collaboration with community-based organisations (such as Dementia Australia and Dementia Support Australia would be beneficial to help reduce pressure on current diagnostic services to provide ongoing post-diagnostic support. A DEMLO role could be utilised as a key link between these services, and facilitate opportunities for further collaboration between community and hospital-based care, which has been shown to be effective in other comprehensive care models (Reuben et al., 2024).

### Evaluation of the Pilot

Ethical approval for the evaluation of the project has been granted by the hospital ethics committee (2023.286). Evaluation of the impact of the DEMLO pilot program will be undertaken via a mixed methods approach, with qualitative interviews conducted with patients and carers, in addition to qualitative/quantitative surveys distributed to staff members of the hospital. These interviews and surveys will be co-designed by members of the research team, with the aim of evaluating consumer and staff satisfaction with dementia care services within the hospital, and any potential improvements made by the DEMLOs. Further quantitative data will also be analysed from the purpose-built DEMLO REDCap database to gather detailed information about service delivery.

## Conclusion

The development and sustainability of a specialist dementia nurse role at a Victorian tertiary hospital encountered several challenges, including limited timeframe, difficulty with integration into existing systems and limited funding. Despite this, the introduction of the DEMLO has resulted in increased person-centred care that encompasses additional pre-diagnostic and post-diagnostic support for people living with dementia. Outcomes of the evaluation of the DEMLO pilot are anticipated to be available later in 2025. If the DEMLO role can assist with improving clinical care outcomes for people living with dementia (and their support persons) and prevent re-admission to other services, it can provide a guide on how to implement these strategies in other relevant services, nationally, internationally, and at scale. This is significant given the predicted exponential growth of older adults living with dementia in the future, and its anticipated economic and public health consequences.

## Data Availability

The data that support the findings of this study are available from the corresponding author, RL, upon reasonable request.*
